# Comparing two data collection methods to track vital events in maternal and child health via community health workers in rural Nepal

**DOI:** 10.1186/s12963-022-00293-4

**Published:** 2022-07-27

**Authors:** Nandini Choudhury, Aparna Tiwari, Wan-Ju Wu, Ved Bhandari, Laxman Bhatta, Bhawana Bogati, David Citrin, Scott Halliday, Sonu Khadka, Nutan Marasini, Sachit Pandey, Madeleine Ballard, Hari Jung Rayamazi, Sabitri Sapkota, Ryan Schwarz, Lisa Sullivan, Duncan Maru, Aradhana Thapa, Sheela Maru

**Affiliations:** 1 Possible, New York, USA; 2 Nyaya Health Nepal, Kathmandu, Nepal; 3 Icahn School of Medicine at Mount Sinai, Arnhold Institute for Global Health, New York, NY USA; 4 Department of Obstetrics and Gynecology, Boston Medical Center, Boston, MA USA; 5 Department of Obstetrics and Gynecology, Boston University School of Medicine, Boston, MA USA; 6 Department of Global Health, University of Washington, Seattle, WA USA; 7 Department of Anthropology, University of Washington, Seattle, WA USA; 8 Henry M. Jackson School of International Studies, University of Washington, Seattle, WA USA; 9 Department of Health Systems Design and Global Health, Icahn School of Medicine at Mount Sinai, New York, USA; 10 Department of Internal Medicine, Icahn School of Medicine at Mount Sinai, New York, NY USA; 11 Department of Pediatrics, Icahn School of Medicine at Mount Sinai, New York, NY USA; 12 Division of Global Health Equity, Department of Medicine, Brigham and Women’s Hospital, Boston, MA USA; 13 Department of Medicine, Harvard Medical School, Boston, MA USA; 14 Division of General Internal Medicine, Department of Medicine, Massachusetts General Hospital, Boston, MA USA; 15 Boston University School of Public Health, Boston, MA USA; 16 Community Health Impact Coalition, New York, NY USA; 17 Department of Obstetrics, Gynecology and Reproductive Science, Icahn School of Medicine at Mount Sinai, New York, NY USA; 18 Possible, Kathmandu, Nepal

**Keywords:** Vital events, Maternal and child health, Community health workers, mHealth

## Abstract

**Background:**

Timely tracking of health outcomes is difficult in low- and middle-income countries without comprehensive vital registration systems. Community health workers (CHWs) are increasingly collecting vital events data while delivering routine care in low-resource settings. It is necessary, however, to assess whether routine programmatic data collected by CHWs are sufficiently reliable for timely monitoring and evaluation of health interventions. To study this, we assessed the consistency of vital events data recorded by CHWs using two methodologies—routine data collected while delivering an integrated maternal and child health intervention, and data from a birth history census approach at the same site in rural Nepal.

**Methods:**

We linked individual records from routine programmatic data from June 2017 to May 2018 with those from census data, both collected by CHWs at the same site using a mobile platform. We categorized each vital event over a one-year period as ‘recorded by both methods,’ ‘census alone,’ or ‘programmatic alone.’ We further assessed whether vital events data recorded by both methods were classified consistently.

**Results:**

From June 2017 to May 2018, we identified a total of 713 unique births collectively from the census (birth history) and programmatic maternal ‘post-delivery’ data. Three-fourths of these births (*n* = 526) were identified by both. There was high consistency in birth location classification among the 526 births identified by both methods. Upon including additional programmatic ‘child registry’ data, we identified 746 total births, of which 572 births were identified by both census and programmatic methods. Programmatic data (maternal ‘post-delivery’ and ‘child registry’ combined) captured more births than census data (723 vs. 595). Both methods consistently classified most infants as ‘living,’ while infant deaths and stillbirths were largely classified inconsistently or recorded by only one method. Programmatic data identified five infant deaths and five stillbirths not recorded in census data.

**Conclusions:**

Our findings suggest that data collected by CHWs from routinely tracking pregnancies, births, and deaths are promising for timely program monitoring and evaluation. Despite some limitations, programmatic data may be more sensitive in detecting vital events than cross-sectional census surveys asking women to recall these events.

**Supplementary Information:**

The online version contains supplementary material available at 10.1186/s12963-022-00293-4.

## Background

Most low- and middle-income countries (LMICs) rely on national representative sample surveys such as Demographic and Health Surveys (DHS) and Multiple Indicator Cluster Surveys for health outcomes data [1, 2]. While national surveys like these offer advantages such as national coverage and standardized data collection, they are inadequate for tracking outcomes with higher geographic or temporal resolution [1, 3]. Timely tracking of vital events and health outcomes is essential for health systems strengthening [4]. Such ‘real-time’ data are unavailable, however, in most LMICs in the absence of comprehensive vital registration systems. Facility-based data are often incomplete and unreliable in rural areas for accurately calculating community-level metrics since many health events occur outside health facilities and are not captured by facility data systems [5, 6].

To accurately and routinely track data at the community level, localized data collection systems are important. In many LMICs, community health workers (CHWs) are increasingly collecting vital events data routinely, including tracking pregnancies, births, and child deaths at the community or village level, as they provide care [7–9]. CHWs are recognized as a promising cadre to help achieve universal health coverage in many low-resource settings [10, 11]. CHWs provide a variety of services in their communities through home visits, including health screening, counseling, and referrals. Mobile technologies assist CHWs in delivering quality services and collecting data in remote and rural areas [12]. With the increase in smartphone users globally, it is increasingly common for CHWs to use mobile technology even in rural settings [13].

Despite the potential of engaging CHWs in timely local data collection and response in LMICs, there have been questions about the quality and completeness of data reported by them for effectively evaluating program and health outcomes. A recent scoping review suggests that community-based vital events reporting is promising but tends to be sub-optimal in data quality and completeness [14]. Studies in Mali, Ethiopia, and Malawi, conducted by the ‘Real-Time Monitoring of Under-Five Mortality’ group, found that vital events data collected routinely by CHWs varied in quality and completeness to accurately assess mortality rates at scale. CHWs’ routine data underreported both births and deaths when compared to household survey estimates [7, 15]. These studies also highlight the importance of further addressing the reliability of using routine data collected by CHWs for priority-setting and decision-making, especially since CHW programs vary widely in supervision, remuneration, and workload [16]. Given that many LMICs have a community workforce that can collect and longitudinally track routine vital events data at the household level, it is important to assess whether these ‘real-time’ data collected by CHWs are reliable for program evaluation.

Like many other LMICs, the vital registration system in Nepal has gaps. For example, only an estimated 42% of births are recorded [17]. It is common to use DHS data, collected every 5 years, to track progress in population health indicators at a national level [18–20]. However, timely tracking of progress in health outcomes at the community level is difficult because of gaps in localized data collection systems. Municipal-level data reporting in Nepal focuses on facility-based indicators and likely misses vital events that take place outside facilities. *Nyaya Health Nepal (NHN)*, a non-governmental organization and its technical partner *Possible*, attempted to address these gaps through household-level data collection embedded within routine care delivery by a trained, salaried, and supervised cadre of CHWs. This CHW model was part of a pilot study conducted in partnership with the Government of Nepal Ministry of Health and Population in two rural districts in Bagmati Province (Province 3) and Far-Western Province (Province 7). The pilot aimed to study the impact of an integrated intervention delivered by this cadre of CHWs on Reproductive, Maternal, Newborn, and Child Health (RMNCH) outcomes [21, 22]. CHWs used a mobile platform (*CommCare*) for counseling, decision support, and simultaneous routine data collection via customized applications and forms [21, 23]. Upon expanding services in a new catchment area, CHWs conducted a one-time household census to identify and enroll married women of reproductive age into the RMNCH care delivery intervention. For each eligible woman, they recorded a birth history for the preceding 2 years from the survey date, with details on child outcomes, birth dates and death dates, if applicable. Following this enrollment period, CHWs regularly visited all enrolled women at home, actively screened for and followed identified pregnancies, and recorded all births in the communities they serve. They also provided postnatal and early childhood care counseling and referrals, monitored the health of children until the age of 2 years every month, and recorded any child deaths [21].

For the RMNCH pilot study, we decided to use routine programmatic data for monitoring and impact evaluation instead of hiring research enumerators, because of resource constraints in the study setting and our goal of improving CHW data collection and its programmatic utilization. Using routinely collected data would also facilitate a sustainable data infrastructure for the CHW program beyond the study period [9]. To assess whether these routinely collected data by CHWs were reliable for evaluating health interventions, we designed a smaller study within the broader RMNCH pilot study. We were limited in our resources to assess the completeness of CHW-collected routine programmatic data with the typically used national census methodology or research data enumerators [24, 25]. Therefore, we sought to assess the consistency of CHW-collected routine programmatic data through comparison with another CHW data collection method, i.e., conducting a household census with birth histories. We compared each vital event (birth, infant death, or stillbirth) from these two methods (programmatic versus census birth history) to address the following questions: Did CHWs identify each birth event equally; classify birth location consistently; identify newborns and infants (up to the age of one year) equally; and classify birth and infant outcomes consistently in both methods?

Here, we present our findings from assessing the consistency of CHW-collected maternal and child health vital events using routine programmatic data versus a census birth history, and implications for using routine data for monitoring and evaluation in a low-resource setting.

## Methods

### Study site

We conducted this study at a site with a total population of approximately 36,000 in the Far-Western Province (Province 7). The study site was part of a larger cluster within a non-randomized implementation research trial on CHW-delivered RMNCH care in Provinces 3 and 7 [21]. Nepal’s hilly Far-Western Province is one of the poorest provinces and was severely affected by the civil war that ended in 2006 [26]. NHN has been providing healthcare in this area since 2008, primarily through a hospital run in partnership with the Government of Nepal. In 2016, NHN started its CHW pilot intervention in the hospital’s immediate catchment area, which spans fourteen wards and comprises the site for this study. CHWs are local women recruited from the catchment area and have completed a tenth-grade education at minimum. They are full-time, salaried employees who receive pre-service training, structured management support, and direct supervision from community health nurses (CHNs) [21]. This program design is largely aligned with the WHO recommendations for effective CHW programs [27]. Each CHW typically serves one ward, a local administrative unit with an average population of 2,500 people in the area [28]. As part of their responsibilities, CHWs go door-to-door to enroll all eligible participants, deliver home-based counseling, conduct basic health assessments, and make referrals for high-risk conditions. CHWs collect data concurrently using customized *CommCare* smartphone applications with inbuilt forms. These serve as both clinical decision support and data collection tools, allowing CHWs to access and update each participant’s longitudinal health record regularly.

**Fig. 1:**
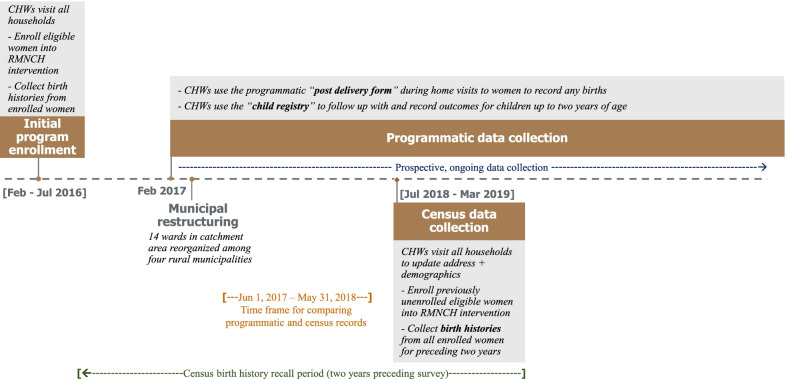
Timeline of programmatic and data collection events

From February to July 2016, CHWs attempted to visit each household in the study site to enroll households for population health surveillance, and married women of reproductive age into the RMNCH intervention (Fig. 1). If families approached for enrollment were unavailable, the CHWs attempted a total of three visits during the enrollment period before designating them as ‘unavailable’. CHWs recorded a 3-year birth history for each eligible woman who provided consent among the 6,384 enrolled households during this period. They obtained birth histories with details on dates of birth and age at death (if applicable) for each child born to surveyed mothers in the preceding 3 years. Following this enrollment phase, CHWs implemented key care delivery components of the RMNCH intervention. These included active pregnancy screening and surveillance via quarterly home visits for all enrolled women, and monthly antenatal and postnatal care home visits for pregnant and postpartum women. In February 2017, home-based care was expanded to include monthly CHW visits for children up to the age of 2 years. Community health nurses frequently accompanied CHWs on their home visits for direct supervision.

**Fig. 2:**
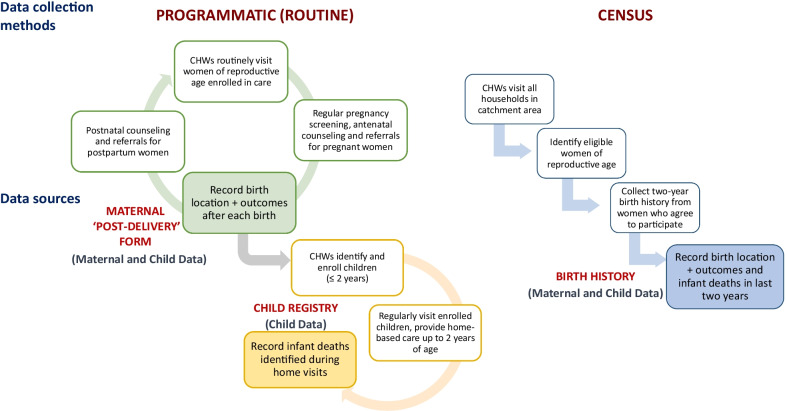
Summary of data collection methods

In March 2017, NHN’s catchment areas were administratively restructured into rural municipalities as part of a broader, nationwide restructuring of local governance following Nepal’s constitutional transition to federalism. During this time, the fourteen wards comprising the study site were reorganized between four distinct municipalities. Following this local restructuring, NHN launched another household census at the study site in July 2018, analogous to the one conducted during the enrollment phase in 2016 (Fig. 1). The primary purpose of this census was for CHWs to update each household’s record using the correct ward and municipality, and to update its address and demographic data within the *CommCare* registry. During this process, CHWs also enrolled any households and eligible women who were not previously enrolled in the RMNCH intervention. Since this household census offered an opportunity for cross-sectional, retrospective data collection, we decided to collect a 2-year birth history to compare vital events with those recorded during routine CHW care delivery (Fig. 2).

### Study population

The study population consisted of married women of reproductive age (15–49 years) and their infants (up to one year in age) in fourteen wards. This was the subpopulation of women enrolled in and defined by the larger RMNCH research study, as is standard in interventions focusing on reproductive health. As such, vital events associated with unmarried women or women outside the specified age window were not included. The 2018 census identified 6,181 married women of reproductive age at the study site. Although the larger RMNCH research study included children up to the age of 2 years, we included data for infants up to the age of one in this study due to practical constraints.

### Data collection

*Routinely collected (programmatic) data:* For this comparison study, we used programmatic data already collected routinely by CHWs while providing care that we used to evaluate the CHW program and the broader RMNCH pilot study. CHWs recorded vital events data on births (including stillbirths) and deaths as they were identified during routine home visits. CHWs completed a programmatic maternal ‘post-delivery form’ in *CommCare* each time a woman gave birth. They recorded information about the birth location type (e.g., hospital, health post, home), birth outcome (stillbirth or live birth), and neonatal deaths among live births (i.e., death within 30 days of birth). Newborn children were enrolled in a separate programmatic ‘child registry’ in *CommCare* for follow-up care. Of note, children (under 2 years old) could have been enrolled in this programmatic child registry even if their mothers were not enrolled in care during pregnancy, or if maternal post-delivery data were unavailable. CHWs then conducted monthly visits to these children and recorded any deaths they identified (Fig. 2). For programmatic records with available child follow-up data, we utilized data from the most recent CHW home visit during the observation period to determine the child’s updated status (‘living’ or ‘death’). Based on the workflow described above, infant deaths could have been recorded in multiple *CommCare* forms, including in the programmatic maternal ‘post-delivery form’ and the regularly updated programmatic ‘child registry’. We extracted data from both forms and merged them to create a composite programmatic record for each mother–child pair, linking mother’s post-delivery data (where available) with each child’s individual follow-up care data in the programmatic ‘child registry’ using a common ‘household ID’ and the birth month and year. We also retained data for children who were enrolled without corresponding programmatic maternal ‘post-delivery’ data, and vice versa.

*Census data:* Our second source of data was the cross-sectional birth history census that CHWs conducted in 2018 while updating household information. We obtained both child mortality and maternal data from the same birth history during the census. Of note, this birth history served as the single source of child data in the census, unlike programmatic data, where infant deaths could be recorded in either the programmatic maternal ‘post-delivery form’ and/or the programmatic ‘child registry.’

NHN’s Mobile Systems Engineer created specific forms within *CommCare* and provided a week-long training for CHWs and CHNs to update house numbers in the household registry and to collect birth history data using a shorter version of the original enrollment census. During household visits, 81 (1%) women of the 6,181 identified were unavailable and 325 (5%) refused to participate. For eligible women who were available and agreed to participate (*n* = 5,775), CHWs recorded information about each birth in the preceding 2 years, including birth location, birth outcomes, and infant deaths (Fig. 2).

In addition to ongoing supervision from CHNs, NHN’s monitoring and evaluation staff and the research team provided continuous technical support to CHWs during census data collection, including conducting regular data quality checks. CHWs completed household renumbering in approximately 4 months between July–October 2018. Pregnancy surveillance (part of the routine RMNCH intervention) was paused during this time to account for the CHWs’ additional workload, while other care delivery services continued as usual. Collecting birth histories took CHWs an additional 5 months and census data collection ended after March 2019. CHWs noted that the delays were primarily because they had to attempt multiple visits for some women who were not at home or had migrated elsewhere when they visited homes for household renumbering.

### Analysis

Accounting for systematic differences in data collection because of the timing and duration of the census described above, we expected both programmatic and census methodologies to theoretically be able to record all births and infant deaths in the 1-year period from June 1, 2017, to May 31, 2018. Birth location was only recorded in the programmatic maternal ‘post-delivery form’ and census data (birth history). Thus, to compare each birth event recorded via maternal data in both sources, we used a unique ID for each eligible woman who gave birth during this period to link and compare records from both sources, counting twins as single births. We then classified each birth as having been recorded in ‘both methods,’ ‘census alone,’ or ‘programmatic alone.’ We categorized each birth location using a binary variable, defining an ‘institutional’ birth as one that took place at any health facility, and a ‘non-institutional’ birth as one that took place at home or on the road. We further assessed if births recorded in both programmatic and census data were classified consistently as ‘institutional’ or ‘non-institutional’ births in both using cross-tabulations.

To assess child outcomes, we merged data on births (including stillbirths) and infants from both the routinely collected programmatic maternal ‘post-delivery form’ and programmatic ‘child registry’, with the census birth history using a combination of a family ID and the birth month and year. We used the family ID to merge records since mother–child pairs were not directly linked in the programmatic database, and used the birth month and year since maternal delivery dates and child birth dates were often off by a few days. In this dataset, we ensured that each birth (collectively from the programmatic maternal ‘post-delivery form' and programmatic ‘child registry’) (including twins) was separated as an individual record to track outcomes. We checked for any duplicates and mismatched records. For any discrepant records identified, we manually verified each data source and cleaned the record, e.g., if a child’s date of birth and mother’s delivery date differed by a month and resulted in two separate observations for the same birth during the data merge. We then classified each birth/infant in the merged dataset as having been identified in ‘both methods,’ ‘census alone,’ or ‘programmatic alone.’ For births (collectively from maternal and child records) that were identified in both data sources, we cross-tabulated the data to check for consistency in classifying outcomes, i.e., ‘stillbirth,’ ‘living,’ or ‘infant death.’ We also conducted a sensitivity analysis for consistency in birth location and child outcomes classification, in which we included records identified by only one source in addition to those identified in both sources (Additional file 1: Table S1 and Additional file 2: Table S2). We conducted all analyses using SAS software version 9.4.

## Results

During the 2018 census, CHWs identified 6,181 married women of reproductive age after updating household records and enrolling newer eligible women into the RMNCH intervention. Of these, 5,775 (95%) agreed to complete the birth history, while 325 (5%) declined. After subsetting census birth history data to births within the 1-year observation period (June 1, 2017, to May 31, 2018), we observed 593 unique births.

After extracting previously collected routine programmatic records (from the maternal ‘post-delivery form’ and ‘child registry’), we limited these data to births within the same 1-year observation period as the census. We observed 646 unique birth records from programmatic maternal ‘post-delivery form’ data alone (counting twins as one record) and 677 unique records collectively from programmatic maternal ‘post-delivery form’ and programmatic ‘child registry’ data.

**Fig. 3:**
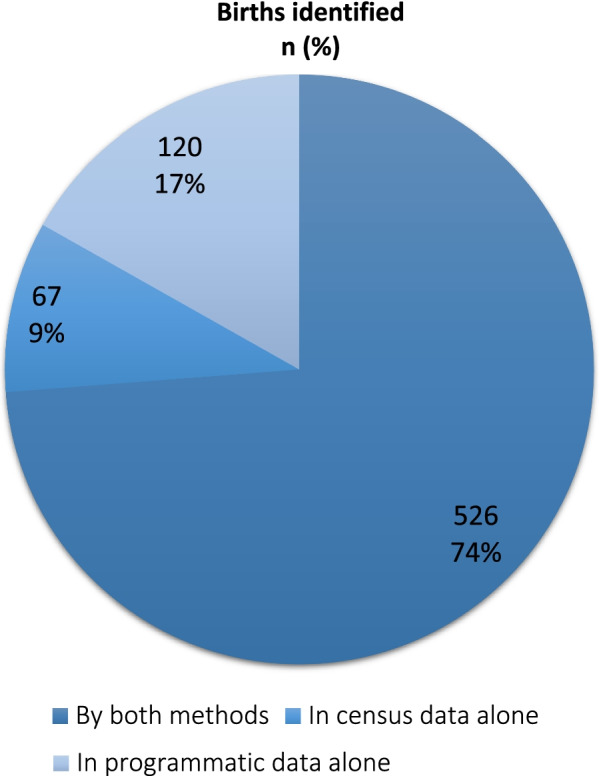
Summary of births identified by census and programmatic (maternal) data, *n* = 713

*Comparing births (from maternal records only) recorded by both methods:* We identified 713 unique births collectively from census data (birth history) and programmatic maternal ‘post-delivery’ records (counting twins as one birth) during the June 1, 2017–May 31, 2018, observation period. Of these, 526 (73.7%) were identified in both data sources, while 67 (9.3%) were identified in census data (birth history) alone, and 120 (16.8%) in programmatic maternal ‘post-delivery form’ data alone (Fig. 3).

*Consistency in birth location classification:* A total of 513 births from census data (birth history) and programmatic maternal ‘post-delivery’ records were categorized consistently as ‘institutional’ or ‘non-institutional’ in both data sources. These comprised 97.5% (95% CI: 96.2%, 98.8%) of the 526 births identified in both sources (Table 1) and 72% (95% CI 68.7%, 75.3%) of all 713 births from maternal records (data not shown in tables or figures).

**Table 1:** Consistency in location classification for each birth identified in *both* census and programmatic data, *n* = 526

	Birth location classification in routine (programmatic) data	
Birth location classification in census data	Non-institutional births *n* (%)	Institutional births *n* (%)
Non-institutional births, *n* (%)	9 (1.7%)	2 (3.8%)
Institutional births, *n* (%)	11 (2.1%)	504 (95.8%)

**Fig. 4:**
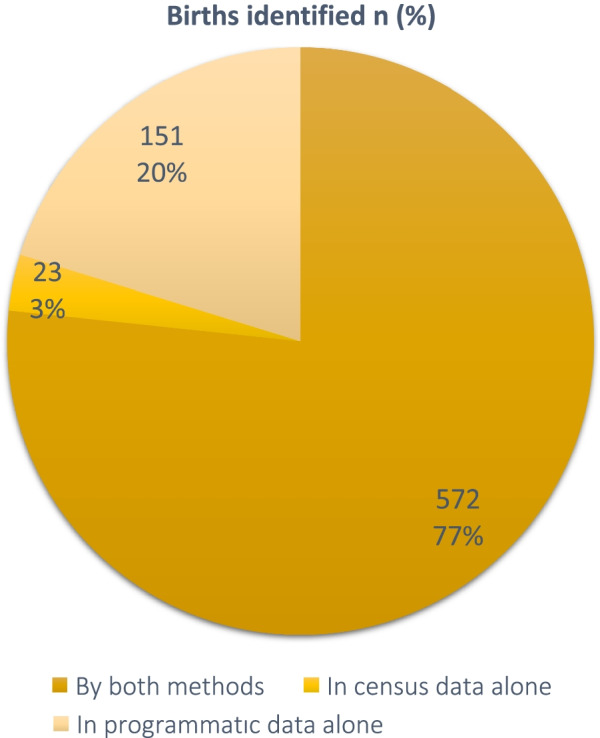
Summary of births identified by census and programmatic (maternal and child registry) data, *n* = 746

*Comparing birth identification in both methods:* We identified 746 unique births collectively from the census (birth history), the programmatic maternal ‘post-delivery form’, and the programmatic ‘child registry’ records between June 1, 2017, and May 31, 2018, in at least one data source. These included infants enrolled in care without corresponding mothers’ post-delivery data (*n* = 73, 9.8%). We identified 572 (76.7%) in both census data and at least one programmatic data source (maternal ‘post-delivery’ and/or ‘child registry’). We identified 151 births (20.2% of total) only in the programmatic (maternal ‘post-delivery’ and/or ‘child registry’) data and 23 (3.1%) only in the census (Fig. 4).

*Consistency in birth outcome classification:* Most birth outcomes (*n* = 566) were categorized consistently as ‘living’ in both programmatic and census data. These comprised 99.4% (95% CI 98.8%, 100%) of the 572 births identified in both sources and 76.3% (95% CI 73.1%, 79.3%) of all 746 births collectively from the census (birth history), the programmatic maternal ‘post-delivery form’ and the programmatic ‘child registry’ records. In contrast, barring two infant deaths that were consistently identified in both programmatic and census data, most were classified inconsistently in both sources, i.e., infant deaths in one source were classified as living or stillbirths in the other data source (Table 2), or were not recorded. Of note, programmatic sources identified five infant deaths and five stillbirths not recorded in census (birth history) data, compared to two infant deaths and one stillbirth recorded in census data but not in programmatic data.

**Table 2:** Consistency in outcome classification for each birth identified in *both* census and programmatic data, *n* = 572

	Birth outcome classification in programmatic (routine) data		
Birth outcome classification in census	Stillbirths *n* (%)	Deaths *n* (%)	Living *n* (%)
Stillbirths, *n* (%)	1 (0.2%)	0 (0.0%)	0 (0.0%)
Deaths, *n* (%)	1 (0.2%)	2 (0.3%)	1 (0.2%)
Living, *n* (%)	0 (0.0%)	1 (0.2%)	566 (99%)

## Discussion

We compared the consistency of data collected by CHWs on vital events that occurred during a 1-year period using two different data collection methods. Programmatic data captured more births from maternal records than a census-based birth history (646 vs. 593). Both approaches seemed to capture some unique births that the other did not—120 births were captured by the programmatic data alone and 67 were captured by the census alone. Programmatic data also captured more birth records collectively from mothers and children than census data (723 vs. 595). We observed very high consistency (> 95%) in classifying vital events among records identified in both programmatic and census data. Further research is needed to draw more inferential conclusions about data completion and quality. However, from our findings, it appears that tracking vital events through CHWs' routine household visits for program monitoring and evaluation, especially in a limited resource setting, may be more sensitive in detecting births and birth outcomes.

One reason for fewer births identified in the census was that 43 women who were enrolled in routine care through the broader RMNCH pilot study did not agree to provide a birth history during the 2018 census. In this setting, the high rate of in- and out-migration poses challenges in care delivery and may explain why some events were missing in the programmatic data [29, 30]. This is consistent with other studies that have identified migration as a potential factor affecting vital events recorded by CHWs [24]. Future studies, especially using qualitative methods, can help explore why some events were missed by the programmatic data, and why women may have declined to provide birth histories during the census.

Although we observed high consistency in birth location classification (institutional and non-institutional) among births identified in both the census (birth history) and programmatic maternal ‘post-delivery form' records (95.8%), there was lower consistency in the sensitivity analysis (72%, Additional file 1: Table S1). However, since non-missing records from both sources tended to be classified as ‘institutional’ births, it is likely that the missing records would also have been classified the same way, which would lead to higher consistency. Of note, we used broad categories and did not compare more granular birth locations within these categories (e.g., ‘hospital’ versus ‘health post’ among institutional births). Further, the high consistency in birth outcome classification (stillbirth, living, and death) for births identified in both the census (birth history), and collective programmatic maternal ‘post-delivery’ and ‘child registry’ data, was almost entirely driven by those in the much larger ‘living’ category. Although both approaches missed some adverse infant outcomes that the other captured, programmatic data identified more deaths and stillbirths than the census. In retrospective data collection, it is common to either fail to report or inaccurately report past events over time [31]. Recall bias may be one reason for missing events from the census method, among other potential reasons including declining participation, and age heaping [32, 33].

Similar studies in other low-resource settings that validated routine data collected by CHWs using different methods have shown varied findings [7]. These studies were conducted as part of the "Real-time Monitoring of Under-Five Mortality" (RMM) project in different countries in Africa [15]. In Mali, the team validated the routine data collected by lay volunteer community-based workers with household census-based full birth history survey data collected by the same volunteer community-based workers. Their study spanned 20 villages with a catchment population of approximately 32,000. Two full-time field coordinators conducted supervision, data verification, and data reviews for feedback loops to support community-based workers. The vital events data that CHWs reported were comparable with the census data and produced similar estimates of under-five mortality [34]. In Malawi, the team compared the expected number of birth and death estimates obtained from routine data collected by health surveillance assistants (HSAs) with rigorous household surveys, collecting complete birth histories for women (aged 15–49 years) in approximately 24,000 households. Two different studies were conducted in two phases at different times, with enhanced supervision and data quality management in the second phase. Each HSA had a supervisor at a health center, who was responsible for field assessment and data quality reviews [24, 35]. HSAs severely underreported births and deaths in both phases despite increased supervision and data quality in the second phase. On average, HSAs underreported births by 44% and under-five deaths by 49% over the study period. Joos et al. [35] cited the challenges of the existing government health systems and high turnover rates of HSAs as the potential reasons for poor data quality. In Ethiopia, a validation study was conducted in two rural zones covering a total population of about 4.4 million. The team compared the vital events data collected by a professionalized home-visiting cadre, health extension workers (HEWs), with data from a household mortality survey. The household mortality survey data were collected using a stratified two-stage cluster sampling design, as part of a larger evaluation that reached approximately 28,000 households. This validation study found severe underreporting of vital events when compared to household survey estimates—HEWs only reported 30% of births and 21% of under-five births causing underestimation in mortality rates. The researchers mentioned the high workload and challenges of supervision in remote areas as some of the potential reasons for low-quality data reported by HEWs [3].

Our findings were more consistent with the study in Mali, where CHWs were able to identify the majority of vital events equally from both approaches. The approaches that the study in Mali and our study used were also similar—both were smaller-scale studies comparing routine data with census-based birth history data collected by CHWs. CHWs in Mali were also able to identify more events from the routine data collection method than the census method, which was similar to our findings [34]. While CHWs in all these studies received some level of supervision and training for data collection, their incentives varied by setting. CHWs in our setting and Ethiopia were salaried, whereas those in Malawi and Mali received some incentives for data collection [3, 24, 34, 35]. However, salaried HEWs in Ethiopia were not able to report complete and quality data due to other local challenges [3]. These findings suggest that a combination of different factors can impact the quality of data reported by CHWs. One key difference between our study and these other studies was our use of a mobile platform instead of paper-based tools or registers. Although CHWs in Ethiopia were part of a salaried and professionalized cadre like those in our study, they used paper-based tools, and research assistants later entered data into a database manually [3]. In contrast, CHWs in our study used a mobile platform with built-in data validation, thereby eliminating incomplete form submissions and the need for manual data entry. In our experience, this was more efficient and less resource-intensive in ensuring better data quality. However, further research can better investigate the complex factors that affect the quality and reliability of CHW routine data collection.

Differences in data quality assurance processes could have contributed to some of the inconsistencies we observed between the two data sources in our study. We began implementing regular data quality checks at the beginning of the census in 2018. Since programmatic data collection preceded the retrospective census data collection and extensive data quality checks, programmatic data quality might not have been as rigorous during the observation period. However, there were some built-in data validations in the *CommCare* forms to reduce anticipated errors during programmatic data collection. Furthermore, since CHWs were updating household data during the census, we found that this introduced some discrepancies in records, such as both old and new household IDs being retained when merging data for analysis. However, this seemed to be the case for only a few records.

There are several limitations to our study. One key limitation was the small study site comprising 14 wards. Infant death is a rare event, and the small numbers we observed limit our ability to make robust inferences using mortality data. Factors such as differences in CHWs’ length of employment, educational level, other competing work priorities, and training may also affect individual data collection. Since the same CHW collected data for her ward using both methodologies in this study, we likely mitigated the effects of these factors when comparing the two approaches. However, this can be a limitation as well: since census data collection followed programmatic data collection, CHWs may have retrieved memories from their care delivery to the same women, which could have influenced the data reported in the census. As has been noted in other studies, CHWs may have experienced a potential conflict of interest since they work to improve health outcomes in their communities, and are also asked to report data on adverse outcomes such as infant deaths [24]. This seems unlikely in our study, however, as the census data also captured fewer births (in addition to fewer unfavorable outcomes) compared to the programmatic data.

In our experience, conducting the census in addition to CHWs’ existing care delivery responsibilities was resource- and time-intensive and may not always be practical in resource-constrained settings. Although pregnancy screening was temporarily halted during the census, CHWs continued to deliver and collect data for services such as antenatal, postnatal, and early childhood care. Census data collection took longer than the expected 4 months to complete, and the community may have experienced survey fatigue. Frequent migration in the setting also posed challenges to completing census data collection within a shorter time period.

Our methods also lacked rigor in collecting mortality and stillbirth data. Although many LMICs commonly use a birth history method for mortality data, it is not always reliable for collecting information on stillbirths and neonatal deaths. There is a high chance of misclassification of self-reported stillbirths and neonatal deaths with this approach [36]. Additionally, stillbirths and miscarriages may often be tied to religious and cultural beliefs in the region [37]. Thus, women may not openly disclose such events in a birth history. This could have caused misclassification or underreporting of mortality and stillbirths in the programmatic data as well. However, this limitation may have been partially mitigated since CHWs belong to the same community as the women they serve, and have built trust with them through continued engagement during care delivery [38]. Future studies should attempt to use a more advanced and in-depth method, such as verbal and social autopsy and participatory analytic methods, and strengthen linkages with government reporting systems, to identify stillbirth and mortality events with greater accuracy [8, 14].

## Conclusions

Salaried, trained, and supervised community health workers are a promising cadre that can help address gaps in routine data collection while delivering care in low-resource settings. Despite some limitations, vital events recorded routinely by CHWs while delivering an integrated RMNCH intervention in rural Nepal were comparable to those collected using a birth history census. Our findings suggest that, despite some limitations, data from routinely tracking pregnancies, births, and deaths over time are promising and seem reliable for timely program monitoring and evaluation at a small scale. Routine programmatic data may even be more sensitive in detecting such vital events than asking women to recall these events. These findings may offer insights to other low-resource settings aiming to use CHW-collected data for timely tracking of progress in health outcomes, in the absence of a comprehensive vital registration system.

## Supplementary Information

**Additional file 1.** **Table S1**: Consistency in institutional birth classification for births identified in the census and programmatic data, n=713

**Additional file 2.** **Table S2**: Consistency in outcome classification for births identified in the census and programmatic data, n=746

## Data Availability

The datasets generated and/or analyzed during the current study will be posted in de-identified format in a publicly accessible data repository and are available from the corresponding author on reasonable request.

## Abbreviations

CHNs: Community health nurses
CHWs: Community health workers
DHS: Demographic and Health Surveys
HEWs: Health extension workers
HSAs: Health surveillance assistants
LMICs: Low- and middle-income countries
NHN: Nyaya Health Nepal
RMNCH: Reproductive, Maternal, Newborn, and Child Health
