# Supplementary material for: Comparing two data collection methods to track vital events in maternal and child health via community health workers in rural Nepal
Source: Popul Health Metr. 2022 Jul 27;20:16. doi: 10.1186/s12963-022-00293-4 (PMC9327361; doi:10.1186/s12963-022-00293-4)
Supplement: Supplementary file 1 — Additional file 1. Table S1: Consistency in institutional birth classification for births identified in the census and programmatic data, n=713 [file 12963_2022_293_MOESM1_ESM.docx]

|  | Birth location classification in routine (programmatic) data | | | |
| --- | --- | --- | --- | --- |
| Birth location classification in census data | **Missing n (%)** | **Institutional births n (%)** | **Non-institutional births n (%)** | **Total n (%)** |
| Missing, n (%) | 0 (0%) | 115 (16.1%) | 5 (0.7%) | 120 (16.8%) |
| Institutional birth, n (%) | 67 (9.4%) | 504 (70.7%) | 11 (1.5%) | 582 (81.6%) |
| Non-institutional, n (%) | 0 (0%) | 2 (0.3%) | 9 (1.3%) | 11 (1.5%) |
| Total, n (%) | 67 (9.4%) | 621 (87.1%) | 25 (3.5%) | 713 (100%) |

**Supplementary Table 1: Consistency in institutional birth classification for births identified in census and programmatic data, n=713**
