# Supplementary material for: Comparing two data collection methods to track vital events in maternal and child health via community health workers in rural Nepal
Source: Popul Health Metr. 2022 Jul 27;20:16. doi: 10.1186/s12963-022-00293-4 (PMC9327361; doi:10.1186/s12963-022-00293-4)
Supplement: Supplementary file 2 — Additional file 2. Table S2: Consistency in outcome classification for births identified in the census and programmatic data, n=746 [file 12963_2022_293_MOESM2_ESM.docx]

**Supplementary Table 2: Consistency in outcome classification for births identified in census and programmatic data, n=746**

|  | Birth/infant outcome classification in programmatic (routine) data | | | | |
| --- | --- | --- | --- | --- | --- |
| Birth/infant outcome classification in census | **Missing  n (%)** | **Deaths n (%)** | **Living n (%)** | **Stillbirths n (%)** | **Total n (%)** |
| Missing, n (%) | 0 (0%) | 5 (0.7%) | 141 (18.9%) | 5 (0.7%) | 151 (20.2%) |
| Deaths, n (%) | 2 (0.3%) | 2 (0.3%) | 1 (0.1%) | 1 (0.1%) | 6 (0.8%) |
| Living, n (%) | 20 (2.7%) | 1 (0.1%) | 566 (75.9%) | 0 (0%) | 587 (78.7%) |
| Stillbirths, n (%) | 1 (0.1%) | 0 (0%) | 0 (0%) | 1 (0.1%) | 2 (0.3%) |
| Total, n (%) | 23 (3.1%) | 8 (1.1%) | 708 (94.9%) | 7 (0.9%) | 746 (100%) |
